# Supplementary figures and images for: Long-Term Effects of Tolvaptan in Autosomal Dominant Polycystic Kidney Disease: Predictors of Treatment Response and Safety over 6 Years of Continuous Therapy
Source: Int J Mol Sci. 2024 Feb 8;25(4):2088. doi: 10.3390/ijms25042088 (PMC10888637; doi:10.3390/ijms25042088)

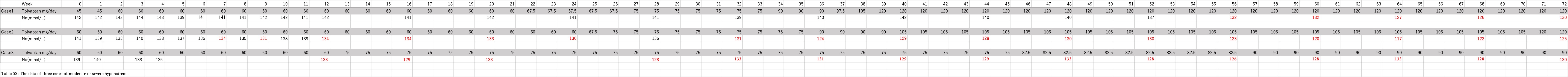

Supplement: Supplementary file 1 [file ijms-25-02088-s001.zip › supplementaly/supplementary2.jpg]

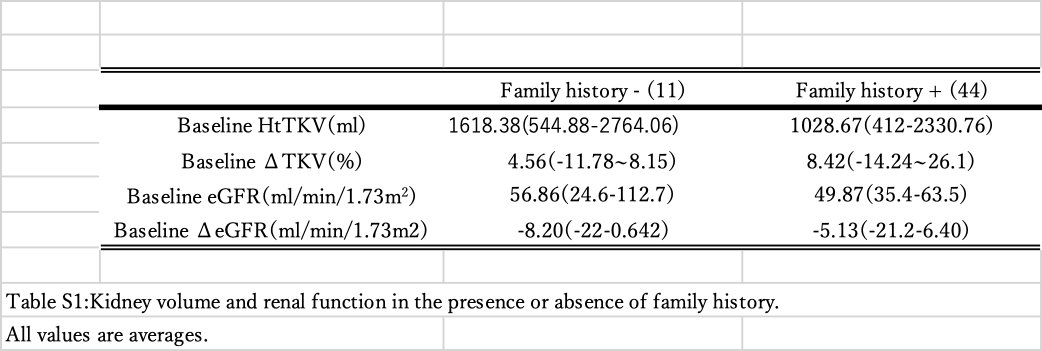

Supplement: Supplementary file 1 [file ijms-25-02088-s001.zip › supplementaly/supplementary1.png]
